# Basic mechanisms and kinetics of pause-interspersed transcript elongation

**DOI:** 10.1093/nar/gkaa1182

**Published:** 2020-12-16

**Authors:** Jin Qian, David Dunlap, Laura Finzi

**Affiliations:** Physics, Emory University, Atlanta, GA 30307, USA; Physics, Emory University, Atlanta, GA 30307, USA; Physics, Emory University, Atlanta, GA 30307, USA

## Abstract

RNA polymerase pausing during elongation is an important mechanism in the regulation of gene expression. Pausing along DNA templates is thought to be induced by distinct signals encoded in the nucleic acid sequence and halt elongation complexes to allow time for necessary co-transcriptional events. Pausing signals have been classified as those producing short-lived elemental, long-lived backtracked, or hairpin-stabilized pauses. In recent years, structural microbiology and single-molecule studies have significantly advanced our understanding of the paused states, but the dynamics of these states are still uncertain, although several models have been proposed to explain the experimentally observed pausing behaviors. This review summarizes present knowledge about the paused states, discusses key discrepancies among the kinetic models and their basic assumptions, and highlights the importance and challenges in constructing theoretical models that may further our biochemical understanding of transcriptional pausing.

## INTRODUCTION

**Figure 1.**
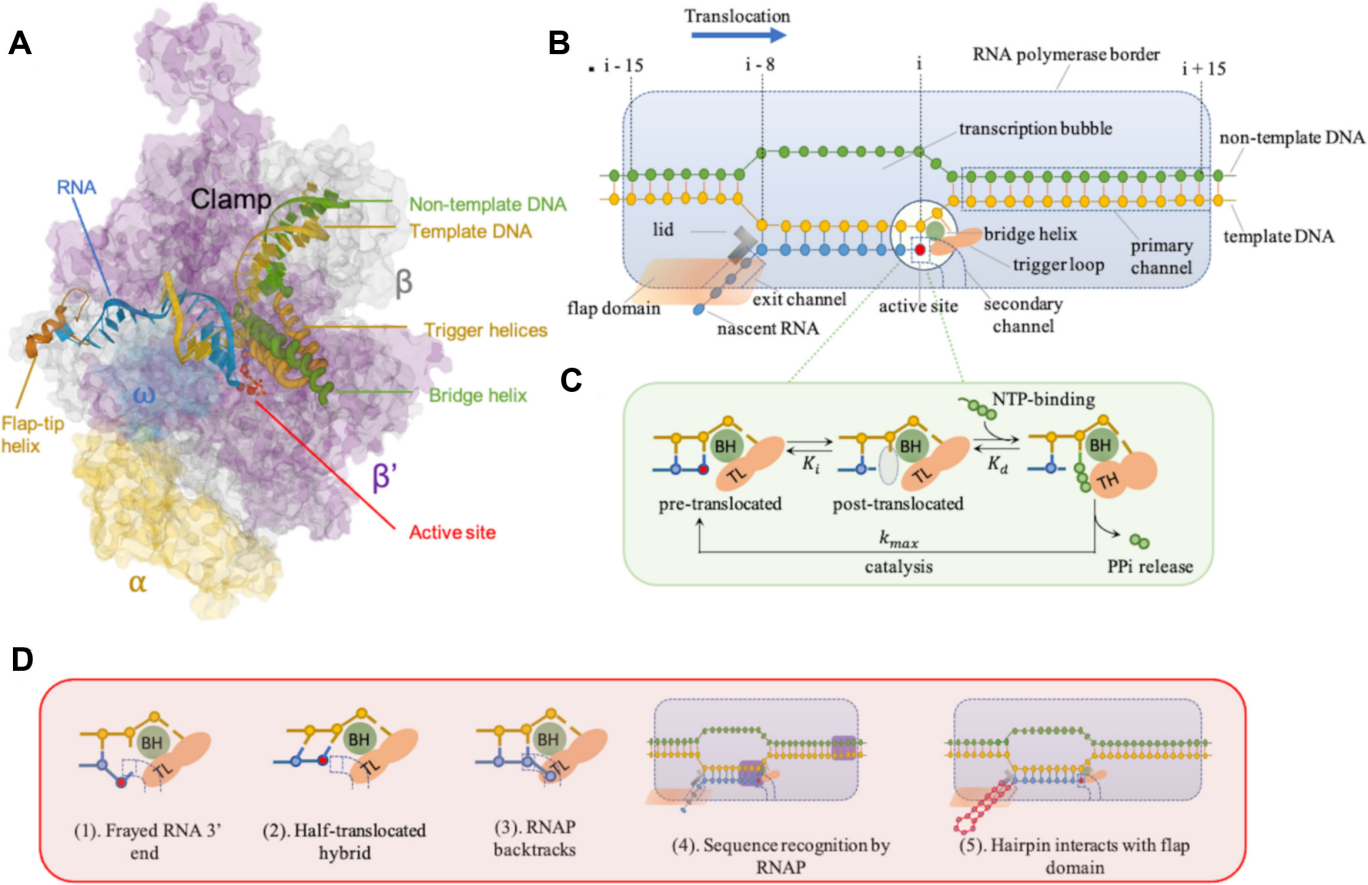
(**A**) Crystal structure of the *Thermus thermophilus* RNA polymerase elongation complex with the bound NTP substrate (PDB: 205J) (3). (**B**) Cartoon of an elongation complex. An elongation complex covers 30–35 base pairs (bp) of DNA, including ∼14 bp of the DNA downstream of the active site, 10–11-bp of the transcription bubble, and nascent RNA which binds the template strand to form an 8–9-bp RNA–DNA hybrid. The RNAP primary channel accommodates the downstream dsDNA; the secondary channel serves as the site for NTP entry and RNA extrusion when RNAP backtracking occurs; the nascent RNA emerges through the exit channel near the flap after the RNA/DNA hybrid strand separates at the lid. The structure is stabilized by the interaction of specific RNAP domains, such as the bridge helix (BH) and trigger loop (TL) that comprise the active center, the lid and flap that interact with nascent RNA. (**C**) A nucleotide addition cycle involves a Brownian ratchet at the active center. RNAP shifts between the pre- and post-translocated registers until an incoming NTP (green) occupies the active site (gray). There, NTP reacts to form a phosphodiester linkage to 3′ -OH group of the growing RNA chain and release inorganic pyrophosphate (PPi). During the process, the TL folds into trigger helices (TH) and positions an NTP for catalysis. (**D**) Off-pathway states proposed to explain elongational pauses include: (1) fraying of the DNA-RNA hybrid at the active site detected by crosslinking, although structural data are not consistent with this; (2) incomplete template DNA strand translocation, with a pre-translocated DNA strand and a post-translocated RNA strand, that precludes NTP addition; (3) RNAP backtracking upstream and extruding the 3′ end of nascent RNA into/through the catalytic site; (4) RNAP recognizing pause signals encoded in DNA/RNA hybrid and/or downstream DNA sequences (purple); (5) a hairpin structure forming in nascent RNA that interacts with the RNAP exit channel and flap domain to cause a global conformational change that disrupts elongation. States 1, 2 and 4 are proposed as elemental pauses. State 3 represents the backtracked paused complex, and state 5 represents the hairpin-stabilized paused complex.

RNA polymerases (RNAPs) constitute a class of molecular motors that consume chemical energy to incorporate nucleotide triphosphates to synthesize RNA. After initial stages of promoter recognition, double strand opening and polymerization of an approximately 8–12 nucleotide long transcript (1,2), RNA elongation is carried out by a transcription elongation complex (TEC) that features a DNA bubble separating upstream and downstream DNA duplexes, an 8–9 nucleotide DNA/RNA hybrid, and an emerging nascent RNA chain. Recent advances in x-ray crystallography and cryo-electron microscopy have revealed further structural features of TEC, such as the trigger loop (TL) and the bridge helix (BH), which are thought to be involved in translocation and proofreading of the 3′ end of the RNA (3–5), the lid and the flap domains that contact the RNA and upstream DNA (Figure 1A and B) (6,7). This structural information, along with experimental data from single-molecule assays, has contributed to our understanding of the mechanism of transcript elongation (8–12). Although some details are still under debate, a Brownian ratchet mechanism, in which TEC forward motion is stochastically generated by thermal fluctuation and then stabilized by molecular pawls (Figure 1C), is favored over other models for the multi-subunit bacterial RNA polymerase. This model gained support mostly because it features a 1-nucleotide translocation step (compared to the power-stroke model in which the step-size is less than 1 nucleotide), and it predicts the experimentally observed force-velocity relationship for transcription under different NTP conditions (13–15).

TECs are processive machines capable of producing long nascent transcripts, yet, transcriptional elongation must be highly regulated in order to respond to abnormal events (e.g. nucleotide misincorporation, transcriptional roadblocks), coordinate co-transcriptional events (e.g. coupling with translation and splicing), and produce biologically meaningful transcripts (e.g. terminating at correct position) (16–19). Regulation of transcription is achieved in large part by pauses that interrupt forward translocation.

During elongation, at every nucleotide coding position, RNA polymerase may follow various kinetically competing reaction pathways. In the event of misincorporation, forward translocation is compromised and various correction pathways, involving backsliding and removal of the ‘wrong’ nucleotide, become kinetically preferred. Alternatively, and very slowly, synthesis past the misincorporated base resumes, resulting in a ‘mutated’ RNA. Finally, transcript termination and release from the transcription complex may also occur. Various pausing mechanisms direct these choices.

Although the functional roles of transcriptional pausing are still under investigation, aberrant pause and release in the human Pol II system has been shown to relate with various human pathologies and reviewed elsewhere (20–23). In prokaryotic systems, transcriptional pauses are well-studied events with lifetimes spread over a broad range, from brief ‘elemental’ pauses (a.k.a. ubiquitous pauses) that last from milliseconds to a few seconds (24–26) to longer-lived pauses that can last for minutes (26–28). Artsimovitch and Landick first proposed that the entry into long paused states begins with the formation of an intermediate, elemental paused state, that can be stabilized mechanistically according to different types of signals and converted into either a ‘backtracked’ or ‘hairpin-stabilized’ paused state (26). These pauses originate from mechanistically different pausing signals and likely have distinct roles in transcription. For example, the backtracked pause is thought to control gene expression at promoter-proximal sites and it is the state leading to correction of misincorporated bases (20,27,29); the hairpin-stabilized pause is thought to guide the folding of leader RNA structures (30). In the recent two decades, single-molecule experimentation and cryo-EM structures of paused TECs have refined our understanding the co-factors that influence the entry into and the escape from paused states (4,11,31,32).

Transcriptional pausing is largely probabilistic, and a vast majority of pausing sites are not 100% efficient (33–35). Thus, the roles of different paused states in transcriptional regulation and the mechanisms that lead to integration of various transcriptional pauses and generation of pause-interspersed transcription are difficult to assess. Nonetheless, many studies have revealed the dynamics of transcript elongation. Here, we review the mechanisms of transcriptional pausing with a focus on the kinetics of the paused states, especially with regard to entry into, and exit from, these states. We hope to provide a mechanistic perspective of how highly regulated transcription develops from a kinetic competition between forward translocation and various paused states of TECs.

### The elementally paused elongation complex (ePEC)

Although the short-lived elemental pauses have been observed in various contexts ranging from ensemble measurements to single-molecule assays, the origin of the ePEC is a subject of debate. In the Brownian-ratchet model, the ePEC stems from the thermodynamics of the RNA–DNA scaffold that permit toggling between the pre-translocated and post-translocated states until NTP binding favors the post-translocated state (Figure 1C) (36,37). This intuitively acceptable explanation was first proposed by Yager & von Hippel and Guajardo & Sousa (38,39). Bai *et al.* used this concept in a quantitative sequence-dependent transcription model which predicted pre-translocated pauses with lifetimes resembling experimentally observed lifetimes of ePECs (40). Indeed, the consensus elements of brief pauses have been identified as a GC-rich segment at the upstream edge of the RNA-DNA hybrid and a pyrimidine at the pause site followed immediately by a G (G-10Y-1G+1), such that G/C at –10 and +1 will disfavor forward translocation relative to the less stable A/T as the Brownian-ratchet model predicts (12,33,34). Although other evidence showed that the elemental pausing also depends on the DNA–RNA hybrid and downstream DNA duplex sequences (33,35,41), the contributions from sequences in the upstream and downstream fork-junctions of the transcriptional bubble demonstrate that the Brownian-ratchet model broadly reproduces what is known about the ePEC.

The Brownian-ratchet model also predicts that the ePEC is an on-pathway intermediate. Experimentally, using core RNAPs that freely transcribe along an optimal pause sequence with a 17-nucleotide downstream segment, Bochkareva *et al.* observed pause events in the pre-translocated state that emphasized the on-pathway mechanism (42). However, this result is disputed by Saba *et al.*, who re-examined kinetic modeling of the pause events along a similar scaffold with an extended downstream segment. They concluded that the obligatory pausing events observed by Bochkareva *et al.* were due to the truncated downstream DNA (41). Overall, the on-pathway mechanism of the ePEC, although intuitively attractive, lacks experimental support.

In fact, experimental evidence seems to place the ePEC off-pathway. Single-molecule experiments have shown that external load on either nascent RNA or motor enzymes has little effect on duration and probability of an ePEC, placing it off the translocation pathway where it is less exposed to mechanical loads (24,43). In experiments with high temporal resolution as short as 1 s, elemental pauses occurred with frequencies well below 100%, implying that the elementally paused state is off the translocation pathway (12,31). Recent cryo-EM structures of the ePEC revealed a post-translocated RNA and pre-translocated DNA forming a tilted RNA/DNA hybrid, along with a rearrangement of the RNAP trigger loop (Figure 1D(2)). This half-translocated state may be an off-pathway state attributable to an elementally paused state and may exhibit longer dwell times than on-pathway intermediates (41,44). Besides the half-translocated register, other conformational arrangements that could inhibit RNAP forward translocation were proposed to explain the formation of the ePEC, such as a frayed 3′ end of the nascent RNA at the active site (Figure 1D(1)), or sequence-dependent nucleic acid and RNA polymerase interactions (Figure 1D(4)) (26,42,45). Backtracked RNAP has been proposed to explain short transcriptional pauses, although this proposition has been disfavored by experimental results (24,46,47).

However, studies on the elongating structure suggest that the translocation step of bacterial RNAP may not act as a simple ratchet driven by NTP-binding affinity. Yin and Steitz, based on the crystal structures of RNAP binding NTP substrates, described a mechanism of translocation in which a helix subdomain of RNAP experiences substantial conformational changes at every cycle of single nucleotide addition. They postulated that RNAPs remain at the pre-translocation position, due to the coordination of the R627 and D537 residues with pyrophosphate, until the rotation of the O helix promoted by the release of pyrophosphate translocates RNAP to the post-translocation position (48). This finding suggests that the release of PPi determines the translocation register rather than the presence of NTPs and contradicts the mechanistic assumptions of most models on transcriptional pauses. In agreement with this finding, a dual-ratchet model was proposed, in which the helix acts as a first reciprocating pawl pushing RNAP forward relative to the nucleic acid scaffold, while the incoming NTP substrate acts as a second stationary pawl preventing RNAP from slipping backward (49).

### Backtracked, paused elongation complex (bPEC)

Backtracking, a reverse motion of RNAP, can induce pausing by extending the 3′ end of a nascent transcript into/through the catalytic site (Figures 1D(3), 2A) (26,28). A subsequent endonucleolytic cleavage event, either intrinsic or induced by accessory factors (e.g. GreA and GreB for *Escherichia coli* and SII/TFIIS for Pol II), can rescue a bPEC (50–52). In *E. coli*, backtracking is known to occur at operon polarity suppressor (*ops*) sites and is responsible for promoter-proximal pausing (26,29). For Pol II in eukaryotes, high G/C content followed by A/T-rich sequence near a DNA promoter is thought to produce unstable RNA–DNA hybridization and polymerase backtracking (53). Using optical tweezers assays, Shaevitz *et al.* reported that backtracking events by RNAP associated with pauses lasting 20 s to >30 min were observed not only at consensus sequences but at locations throughout DNA templates (27).

**Figure 2.**
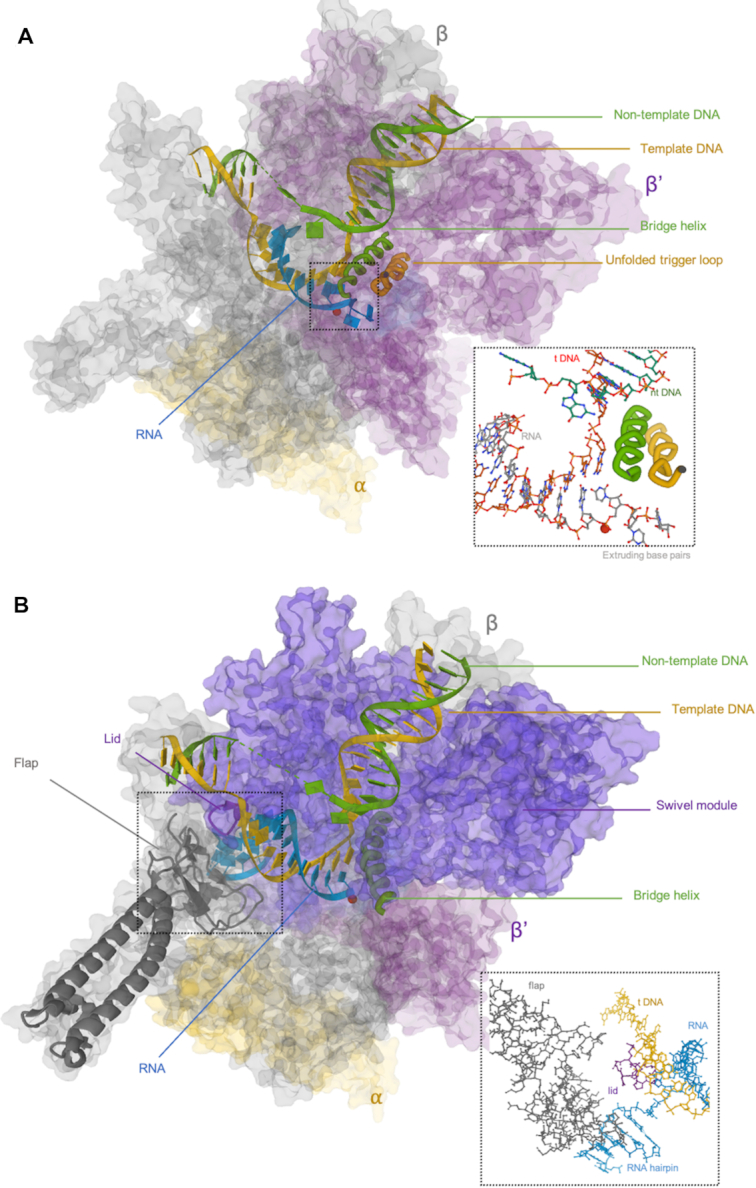
(**A**) Crystal structure of a backtracked *E. coli* RNA polymerase elongation complex (PDB: 6RI9) (86). Notice the RNA (cyan) bases extruded beyond the catalytic site in the enlargement. (**B**) Crystal structure of a hairpin-stabilized *E. coli* RNA polymerase elongation complex (PDB: 6ASX) (44). Notice the flap domain (gray) interacts with the hairpin (cyan) and stabilizes the swivel module (purple).

The formation of bPECs is force sensitive, and the pathway leading to bPECs is relatively well understood. Assisting or opposing loads respectively inhibit or facilitate the occurrence of a bPEC at a pause site. This indicates that bPECs are energetically stable states produced by reverse translocation of RNAP (54). Saba *et al.* proposed that an ePEC could equilibrate rapidly among the pre-translocated, half-translocated and one-base-pair backtracked states, given that the energy associated with a single base pair is a minimal barrier (41). Other work using high-resolution optical trapping assays also demonstrated the ease with which a one-base-pair bPEC forms (37,55). More extended backtracking is less frequently observed, but is characterized by longer dwell times, so the formation of deeper bPECs likely involves greater activation barriers. Also, there is evidence that a conformational change might be associated with backtracking and drive the bPEC into a state resistant to rescue by external loads and RNA cleavage events (45,56,57).

Recovery from backtracking is thought to occur by either 1D diffusion or cleavage of RNA blocking the catalytic site. An optical trapping assay on RNAP II has shown that the distribution of backtracked pauses of less than 10 s follows a *t*^−3/2^ power law, implying backtracked RNAP II diffuses following a 1D unbiased random walk with one nucleotide steps in the absence of RNA cleavage events (54). Lisica *et al.* reported that the choice of the recovery mechanism is determined by a kinetic competition between the random walker and the RNA cleavage event (58). A bPEC in a shallow energetic trap, such as one-base-pair, tends to recover by 1D diffusion, while a bPEC in a deep energetic trap is more likely to recover through RNA cleavage. In recent work using high-throughput magnetic tweezers, Janissen and colleagues supported this by showing that recovery of PECs backtracked by >4-bp is predominantly achieved by intrinsic cleavage, in the absence of cleavage factors that significantly facilitate recovery (57).

### Hairpin-stabilized paused elongation complex (hsPEC)

An important class of long-lived pausing signals is encoded in RNA secondary structures (Figures 1D(5), 2B). A nascent RNA structure, perhaps a pseudo knot or a hairpin, can interact with the flap domain near the exit channel and inhibit nucleotide addition in the active site 65 Å away (6,59–61). According to an allosteric model supported by cryo-EM reconstruction, upon hairpin-flap interaction, RNAP adopts a global conformational rearrangement that stabilizes a swivel module and prevents trigger loop folding, disrupting the active nucleotide addition cycle (7,44). Transcriptional factor NusA is known to enhance hairpin-mediated pausing by providing a positively charged cavity in the RNAP exit channel for the formation of RNA secondary structure and the stabilization of RNA–RNAP interaction (7). Note that an RNA hairpin also helps disrupt the RNA–DNA hybrid during intrinsic transcriptional termination. Hairpins may induce similar conformational rearrangements of polymerases in both hairpin-stabilized pausing and intrinsic termination (26,62–64).

Although the mechanisms underlying the hsPEC are not fully understood, many features of the hsPEC have been revealed experimentally. Recently, experimental data have separately shown that the RNAP flap domain, trigger loop, and RNA hairpin are indispensable elements for the formation of a hsPEC, in agreement with the mechanism suggested by the cryoEM hsPEC structure (55,65–67). A four-nucleotide separation between the hairpin and the RNA–DNA hybrid is optimal for the formation of an hsPEC. Decreasing this spacer to 2 nt substantially reduces pausing (6). In addition, the stability and size of the hairpin affect the formation of the hsPEC. Chauvier *et al.* found that a stable stem favors formation while the size of the end-loop is less important (67). However, overall dimensions are important, and Toulokhonov *et al.* found that hsPEC formation drops substantially for RNA hairpins with longer stems. Surprisingly, an artificial hairpin formed by hybridization of an oligonucleotide to the nascent transcript cannot mimic the effect of the nascent RNA hairpin (68).

### Kinetics of pause-interrupted transcription

**Figure 3.**
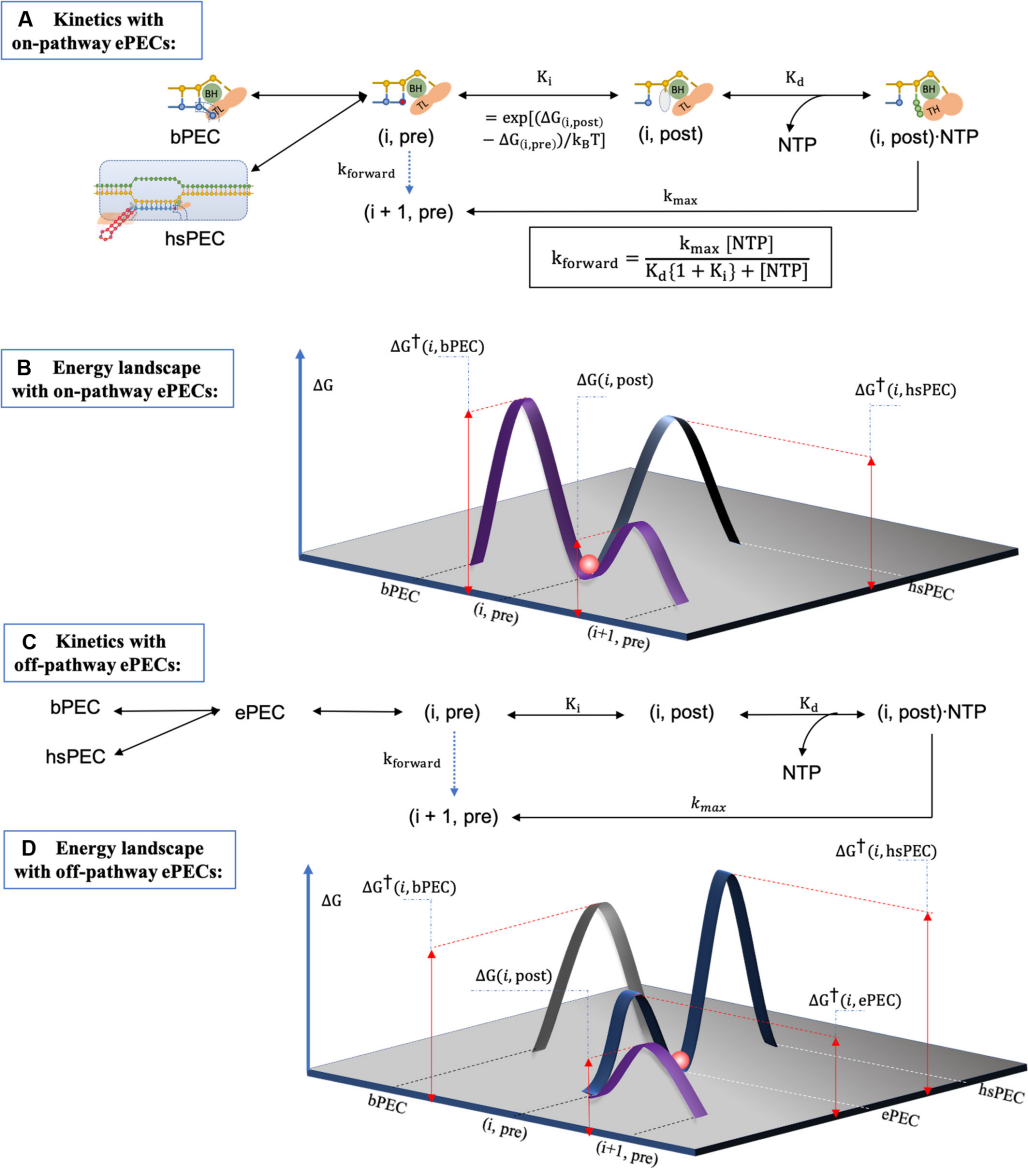
(**A**) A kinetic scheme of pause-interrupted elongation, with an on-pathway elementally paused complex (ePEC). The equilibrium constant *K*_i_ is determined by the free energies of the pre- and post-translocated configurations. *K*_d_ is the NTP-dependent dissociation constant, and *kmax* is the NTP-specific reaction rate of an irreversible step including NTP catalysis, PPi release and possibly other steps to catalyze phosphodiester bond formation between the 3′ end of the nascent transcript and an incoming NTP. The overall forward rate from the (*i*, pre) state to the (*i* + 1, pre) state, indicated by the dashed arrow as a virtual connection, is given by the Michaelis-Menten equation with parameters *K_i_*, *K*_d_ and *k*_max_. (**B**) A qualitative energy landscape for the kinetic scheme in A. The elementally paused complex (red sphere) and hairpin-stabilize complex are translocationally similar to the (*i*, pre) state. The backtracked, paused complex is translocationally distinct from both the ePEC and the (*i*, pre) state. (**C**) A kinetic diagram with an off-pathway ePEC state that is conformationally different from the pre-translocated complex state and can further convert to the bPEC or hsPEC states. (**D**) A qualitative energy landscape associated with the kinetic scheme described in C.

The Brownian-ratchet kinetic model of the active elongation pathway consists of a translocation step, an NTP binding step, and a rate-limiting NTP nucleation step (Figures 1C and 3A) (14,40,69). The overall kinetics is described by the Michaelis-Menten equation.

(1) $$\begin{equation*}{k_{{\rm{foward}}}} = \frac{{{k_{{\rm{max}}}}{\rm{\ }}\left[ {{\rm{NTP}}} \right]}}{{{K_d}\left( {1 + {K_i}} \right) + \left[ {{\rm{NTP}}} \right]}}\ \end{equation*}$$

where *K_i_* is the equilibrium rate between the pre- and post-translocated state of TEC at position *i*, *K*_d_ is NTP-specific parameters for NTP dissociation and *k*_max_ is the reaction rate of an irreversible step including NTP catalysis, PPi release and phosphodiester bond formation (Figure 3A). To measure the values of the NTP-specific parameters in Equation (1), Bai *et al.* recorded the transcriptional rates of *E*. *coli* RNAP along DNA segments lacking one of the four nucleotides and reported the fitted parameters in which *K*_d_ ranged from 7 μM for CTP to 62 μM for GTP and *k*_max_ ranged from 18 s^–1^ for UTP to 50 s^–1^ for ATP (14). Force (*F*) perturbations would be expected to modulate the kinetics of the equilibrium of translocation step by *K_i_* = exp[(Δ*G*(*i*, post) − Δ*G*(*i*, pre) − *F*δ)/*k*_B_*T*], where δ is a 1 nucleotide step by the ratchet-like TEC. However, this simplest ratchet model does not address the roles of the folding and unfolding of the BH and the TL, thus may not fully represent the mechanistic origin of transcriptional kinetics. A more universal and detailed model that considered the folded/unfolded states of bridge helix and trigger loop further classified six interconnected states within a nucleotide addition cycle (70).

The ePEC, bPEC and hsPEC were depicted as thermal states branching from the active elongation complex. As illustrated in Figure 3, forward elongation dynamics result from the probabilities of transition across activation barriers connecting the paused and active states (71). This mechanism suggests that an elongation complex favors pausing at low free energy positions. Indeed, Ruckenstein and colleagues simulated the occurrence of pausing by evaluating the local minimum in free energy landscape over a finite region in the vicinity of translocation position (72). For the formation of the ePEC, an on-pathway Brownian-ratchet interpretation leads to pre-translocated pauses at sites where ΔG(i, post) is much greater than Δ*G*(*i*, pre) (Figure 3A) (14,40). In contrast, if the ePEC is considered an off-pathway intermediate, entry into the ePEC can be achieved by either forward translocation from the (*i* – 1, post) state, or by inactivation from the (*i*, pre) state. Indeed, the latter (Figure 3C) is supported by previous cross-linking experiments (6,31,34,73) and the fact that stabilization of post-translocated TECs would reduce the efficiency of elemental pause sites. Ruckenstein and colleagues reported that in models considering solely the pre-translocated pausing (with an energy landscape shown in Figure 3B), transcription tends to arrest quickly and the frequency of pausing is below that observed experimentally, while addition of an intermediate state preceding the entry to the backtracked pause (i.e. an off-pathway, elementally paused state) can resolve these problems (69). Also, they reported that the pre-translocated pause still comprises the majority of short pauses even if an intermediate state is included in the model. These findings indicate that pre-translocated and intermediate paused states may indeed coexist (and form an energy landscape as shown in Figure 3D) but remain indistinguishable in experimental data.

The entry to the backtracked pause is usually modeled as an off-pathway event due to the upstream movement of RNAP across an energetic barrier. Although the energetic barriers of deeper backtracked complexes are likely larger than those of one-base-pair backtracked complexes, they are taken as constant in most cases (40,46). Backtracking recovery is usually simulated as a force-biased continuous or discrete random walk, where the backtrack dwell time is described as a first-passage time to reach an active registry (40,74). RNA cleavage functions as a stochastic resetting process that competes with the random walker (75). Moreover, the secondary structure of nascent transcripts is often considered a barrier that precludes deep backtracking (72,76). For *E. coli* genome pause sites, Bai *et al.* fitted a backtracking barrier of 46.2*k*_B_*T* and incorporated only a 1D diffusive recovery pathway, while Douglas *et al.* estimated the barrier as a normal distribution centered at 5k_B_T and incorporated a cleavage pathway for backtracks of <10 nucleotides (40,77). Despite significantly different energy barriers and recovery pathways, both models accurately predicted pause positions and dwell time distributions, indicating the difficulty in determining a biologically accurate model for the manifold transcription process.

As a counterpart to bPECs, hypertranslocated PECs, in which a TEC may translocate ahead without concomitant 3′ RNA extension, were considered in several models. Hypertranslocated pauses have been reported experimentally and were thought to be the rate-limiting step for hsPEC until a more plausible mechanism was revealed from cryo-EM structures (26,78). The approach to model hypertranslocated pauses is similar to that of the backtracked pauses, with an energetic barrier to entry and escape through 1D thermally-driven diffusion (40,77).

The hsPEC is a class of transcriptional pauses for which the kinetics are least understood. No reported models depict the kinetics of the entry to and the escape from the hsPEC, due in large part to the difficulty of quantitatively detecting interactions between RNA and RNAP residues. Indeed, Dalal *et al.*, who used optical tweezers to pull directly on the nascent mRNA and inhibit folding during transcription, found that the kinetics of pausing were not affected by the perturbation (43). Future single-molecule experiments with lesser mechanical perturbations to the polymerase than used so far may be necessary to reveal hsPECs which nonetheless may comprise a minor fraction of paused states.

## DISCUSSION

Currently, our understanding of the kinetics of transcript elongation trails our understanding of the mechanics. Although cryo-EM structures of paused TECs gave insight into the mechanisms of distinct pausing signals, these static snapshots cannot reveal the kinetics of pause states. For kinetics, we rely on single molecule experiments that reveal the dynamics of transcript elongation in conjunction with modeling that can decompose the overall dynamics into contributions from individual components. A Brownian ratchet model accurately reflects the rate and the force-dependence of the processive elongation observed in experiments. However, the kinetics of paused states are less clear: (i) in different proposed models, the ePEC are represented as either on-pathway or off-pathway states, and the models do not seem to completely fit the mechanistic origin of the ePEC; (ii) kinetics of the bPEC are well characterized, but fit-determined parameters vary according to experimental constructs; (iii) and finally, experiments to analyze the kinetics of the hsPEC are difficult. Overall, the difficulty in characterizing the kinetics of the paused states arises from distinguishing the observed pauses experimentally, especially for the elemental and hairpin-stabilized PECs which cannot be distinguished translocationally in single-molecule transcription assays. Even for bPECs, which are characterized by reverse motion of TECs, efficient identification requires base-pair resolution and high signal-to-noise ratio experiments that are difficult to achieve in most laboratories.

The Brownian-ratchet model predicts the configuration of a transcription bubble to be a subtle but critical element that could significantly affect the kinetics. Nucleotides 1–2 positions proximal to the catalytic site and the unpaired nucleotides at the edges of the bubble are important, because they chiefly determine the relative stability of the pre- and post-translocated states, and hence the possibility of entering a paused state. Many models employ a fixed-length transcription bubble and a fixed-length DNA/RNA hybrid (40,77,79). These assumptions conflict with changes in the size of the transcription bubble detected experimentally and may introduce thermally unfavorable bubble configurations that might change spontaneously. A statistical mechanics approach was implemented in some model constructs to account for the variation in bubble/hybrid size (72).

The Brownian-ratchet model of RNAP transcription was proposed 30 years ago and has been subsequently refined. However, a significant defect of the model is a probable over-simplification of the real transcription mechanism, such as neglect of a potential allosteric nucleotide binding site that the elongation complex may contain, as proposed by Foster *et al.* (80). In addition, the effects of transcriptional modulators are overlooked in most models, and none address heterogeneity of pausing among species. For example, in *E. coli*, NusG is an anti-pausing factor that could stimulate forward translocation and prevent RNAP backtracking, while in *Bacillus subtilis*, NusG induces pausing by shifting RNAP to the post-translocation register (26,81–84). Another example is that *E. coli* RNAP recognizes a well-characterized hairpin-stabilized *his* pause site, while *B. subtilis* RNAP and mammalian Pol II do not respond to this hairpin-mediated signal (26,85).

Despite the limitations, models of transcript elongation have produced new insights about the kinetics of mechanistically identified paused states. For example, in modelling the distribution of pause times of bacterial TEC, Janissen *et al.* identified three interconnected pause states, two of which appear to be backtracked PECs. They found that the recovery from one occurs 20 times slower than that from the other and cannot be accelerated by cleavage factor GreB (57). Therefore, they postulated that a bPEC could undergo conformational changes to enter a longer-lived RNA-cleavage-resistant state. Douglas *et al.*, by comparing models with and without the intermediate state, backtracking, hypertranslocation and RNA folding, found that these factors are not necessary for predicting the locations and frequencies of pauses (77). Thus, they concluded that occurrence of pauses is chiefly facilitated by the relative stability between the pre- and post-translocated states, while the off-pathway events only serve to extend the pauses.

In the future, we hope to see a unified kinetic model of transcript elongation that concurs with our biochemical understanding of the effects of other factors on paused complexes and predicts the experimentally characterized pause sites and pause frequencies. An immediate difficulty is the construction of distinctive models for the hsPEC and other mechanistically similar states and experimental methods with which to detect them. Moreover, such models must be consistent with the effects of transcriptional modulators that accentuate particular mechanisms of elongation and pausing from prokaryotes to eukaryotes.
